# Human mesenchymal stem cells are resistant to UV-B irradiation

**DOI:** 10.1038/s41598-019-56591-9

**Published:** 2019-12-27

**Authors:** Ramon Lopez Perez, Jannek Brauer, Alexander Rühle, Thuy Trinh, Sonevisay Sisombath, Patrick Wuchter, Anca-Ligia Grosu, Jürgen Debus, Rainer Saffrich, Peter E. Huber, Nils H. Nicolay

**Affiliations:** 1 Department of Molecular Radiation Oncology, German Cancer Research Center (dkfz), 69120 Heidelberg, Germany; 2 Department of Radiation Oncology, Heidelberg University Hospital, 69120 Heidelberg, Germany; 3 Department of Radiation Oncology, University of Freiburg - Medical Center, 79106 Freiburg, Germany; 4 Institute of Transfusion Medicine and Immunology, German Red Cross Blood Service Baden-Württemberg - Hessen, Medical Faculty Mannheim, Heidelberg University, Heidelberg, Germany

**Keywords:** Mesenchymal stem cells, Skin diseases, Skin cancer

## Abstract

Albeit being an effective therapy for various cutaneous conditions, UV-B irradiation can cause severe skin damage. While multipotent mesenchymal stem cells (MSCs) may aid the regeneration of UV-B-induced skin injuries, the influence of UV-B irradiation on MSCs remains widely unknown. Here, we show that human MSCs are relatively resistant to UV-B irradiation compared to dermal fibroblasts. MSCs exhibited higher clonogenic survival, proliferative activity and viability than dermal fibroblasts after exposure to UV-B irradiation. Cellular adhesion, morphology and expression of characteristic surface marker patterns remained largely unaffected in UV-irradiated MSCs. The differentiation ability along the adipogenic, osteogenic and chondrogenic lineages was preserved after UV-B treatment. However, UV-B radiation resulted in a reduced ability of MSCs and dermal fibroblasts to migrate. MSCs exhibited low apoptosis rates after UV-B irradiation and repaired UV-B-induced cyclobutane pyrimidine dimers more efficiently than dermal fibroblasts. UV-B irradiation led to prolonged p53 protein stability and increased p21 protein expression resulting in a prolonged G2 arrest and senescence induction in MSCs. The observed resistance may contribute to the ability of these multipotent cells to aid the regeneration of UV-B-induced skin injuries.

## Introduction

UV-B irradiation is an effective therapy for the treatment of various cutaneous conditions including psoriasis, atopic dermatitis and mycosis fungoides^1–4^. The successful application of UV-B irradiation in autoimmune skin disorders illustrates the immunosuppressive capacity of UV-B light. UV-B reduces the antigen-presenting activity of epidermal Langerhans cells, induces apoptosis in T cells and results in the release of immunosuppressive factors by keratinocytes^5–7^. Beyond the therapeutic features, UV-B light exhibits carcinogenic potential and can cause long-term skin damage^8^. The carcinogenicity is mainly related to direct photochemical DNA damage, especially pyrimidine dimerization^9^. The most prevalent products of pyrimidine dimerization are cyclobutane pyrimidine dimers (CPDs) and 6-4 pyrimidine-pyrimidone (6-4PP) photoproducts, whereby CPDs account for 80% of all UV-B-induced DNA mutations^10^. Furthermore, generation of reactive oxygen species (ROS) through UV-B light causes secondary damage to cellular DNA, proteins and lipids^11^.

Mesenchymal stem cells (MSCs) were first identified in bone marrow in the 1960s, but have since been identified in many other tissues including adipose tissue, umbilical cord and skin^12–16^. Comparison of study results is hindered by the heterogeneity in the isolation and expansion of these multipotent stem cells, wherefore the International Society for Cellular Therapy has proposed minimal defining criteria, especially the cells’ capability to adhere on plastic surfaces, their expression of distinct surface markers and their ability to differentiate into osteoblasts, adipocytes and chondroblasts^17^.The regenerative ability of MSCs has been shown in various preclinical and clinical investigations and is attributed both to their differentiation potential and their paracrine effects^18–20^. Recently, secretion of immunomodulatory cytokines, growth factors and exosomes is believed to constitute the leading mechanism for MSCs-based tissue repair^21,22^.

MSCs have been demonstrated to protect dermal fibroblasts from UV-B-induced oxidative stress and to attenuate UV-B-induced skin damage^23,24^. Secretory factors of MSCs resulted in increased collagen levels, thereby reducing UV-B-related wrinkles^24^. Furthermore, promising results regarding MSC-based therapies of autoimmune skin diseases such as psoriasis and atopic dermatitis have been obtained^25,26^. As UV-B irradiation belongs to the standard treatments in these autoimmune diseases, a profound knowledge about the effects of UV-B on MSCs is crucial to identify potential interactions between UV-B treatment and MSC-based therapies. While the effects of ionizing radiation and chemotherapeutic agents on MSCs have been investigated in depth, the interaction between UV-B irradiation and MSCs remains mostly unknown^27–29^.

Here, we elucidated the influence of UV-B irradiation on the survival and functional characteristics of MSCs compared to dermal fibroblasts. Additionally, we examined cellular response mechanisms of MSCs after UV-B irradiation including the repair of UV-induced DNA damage.

## Results

### MSCs are more resistant to UV-B irradiation than dermal fibroblasts

**Figure 1:**
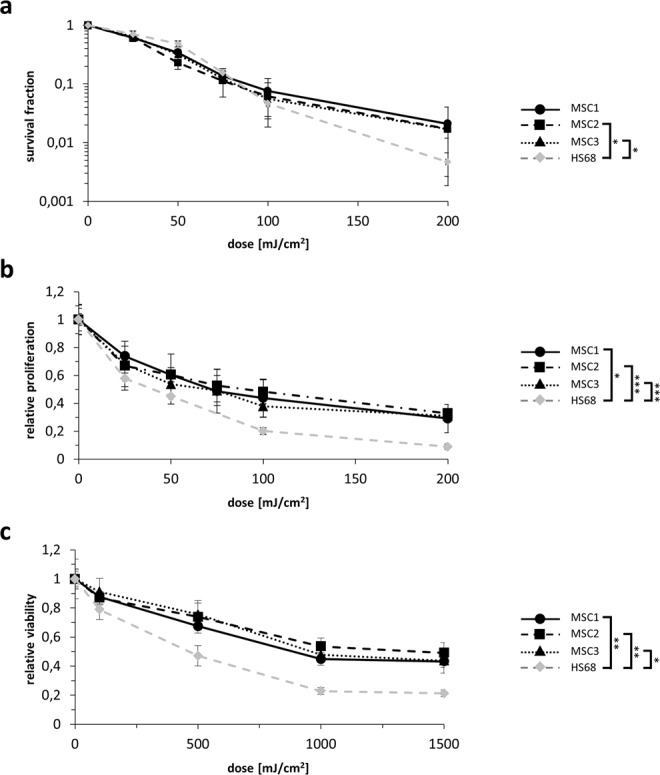
MSCs are more resistant to UV-B irradiation than dermal fibroblasts. (**a**) Clonogenic survival assays for human MSCs and HS68 dermal fibroblasts. (**b**) Relative proliferation at 96 hours after UV-B treatment of MSCs and dermal fibroblasts. (**c**) MTS assays showing metabolic viability at 96 hours after UV-B irradiation using doses up to 1500 mJ/cm^2^. *P < 0.05, **P < 0.01, ***P < 0.001. Two-sided Student’s t-tests at 200 mJ/cm^2^ (clonogenicity and proliferation assays) and 1500 mJ/cm^2^ (viability assays) were used. Mean ± standard deviation is shown, n = 3.

Sensitivity of human MSCs and dermal fibroblasts to different doses of UV-B irradiation was assessed using clonogenic proliferation and viability assays. MSC2 and MSC3 exhibited elevated clonogenic survival levels compared to dermal fibroblasts after UV-B irradiation (*P* < 0.05 for MSC2 and MSC3, Student’s two-sided t-test at 200 mJ/cm^2^), while there was only a non-significant trend towards increased UV-B resistance for MSC1 (*P* = 0.67) (Fig. 1a). Both proliferation and metabolic viability assays revealed higher survival rates of MSCs than HS68 fibroblasts after UV-B exposure. Relative proliferation rates of MSCs after 200 mJ/cm^2^ UV-B irradiation were found increased by more than factor 3 compared to HS68 fibroblasts (*P* < 0.05 for MSC1, *P* < 0.001 for MSC2 and MSC3) (Fig. 1b). After UV-B irradiation with doses up to 1500 mJ/cm^2^, MSCs remained considerably more viable than dermal fibroblasts (*P* < 0.01 for MSC1 and MSC2, *P* < 0.05 for MSC3) (Fig. 1c).

### UV-B treatment leads to heterogeneous results regarding MSCs adhesion ability

**Figure 2:**
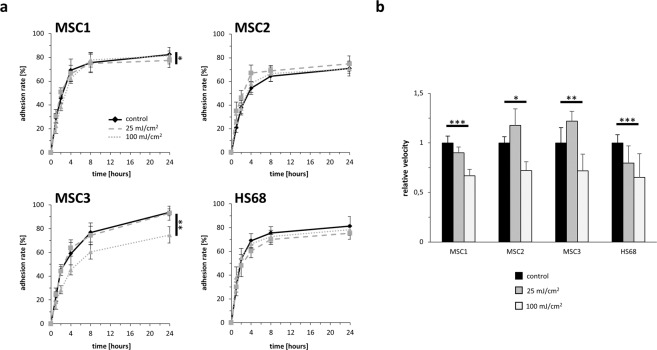
UV-B treatment does not abrogate the adhesion ability but impairs the velocity of MSCs. (**a**) Relative adhesion rate of MSCs and HS68 after UV-B irradiation with 25 mJ/cm^2^ or 100 mJ/cm^2^. (**b**) Average cellular velocity in untreated and UV-B irradiated MSCs and dermal fibroblasts. *P < 0.05, **P < 0.01, ***P < 0.001. Data are mean ± standard deviation, n = 4.

MSC adherence was examined over a period of 24 hours after UV-B exposure. While there was no delay in cellular attachment of MSCs, number of attached cells differed between unirradiated and UV-B-irradiated cells in MSC1 and MSC3 24 hours after UV-B irradiation. MSC1 cells were shown to exhibit lower adhesion rates 24 hours after low-dose (25 mJ/cm^2^) but not after high-dose UV irradiation (100 mJ/cm^2^) (*P* < 0.05). In contrast, irradiation with 100 mJ/cm^2^ resulted in a significant reduction of adhesion in MSC3 (*P* < 0.01), whereas low-dose irradiation with 25 mJ/cm^2^ led to comparable adhesion rates between irradiated and untreated cells. MSC2 showed no changes in their cellular attachment rates 24 hours after UV irradiation (*P* = 0.29 for 25 mJ/cm^2^, *P* = 0.95 for 100 mJ/cm^2^) (Fig. 2a). Both low-dose (25 mJ/cm^2^) and high-dose (100 mJ/cm^2^) UV-B irradiation did not reduce the adhesion ability of HS68 fibroblasts (*P* = 0.25 for 25 mJ/cm^2^, *P* = 0.52 for 100 mJ/cm^2^).

### UV-B irradiation reduces cellular velocity of MSCs

Cellular movement was investigated over a period of 35 hours after UV-B irradiation using time-lapse microscopy. While 25 mJ/cm^2^ UV-B irradiation did not influence cellular motility of MSCs, exposure to 100 mJ/cm^2^ UV-B reduced the average velocity of all analyzed MSC preparations (*P* < 0.001 for MSC1, *P* < 0.05 for MSC2, *P* < 0.01 for MSC3) (Fig. 2b). After 100 mJ/cm^2^ UV-B irradiation, the average MSC velocity significantly decreased to values ranging between 66.8% and 72.0% of untreated controls. Notably, HS68 fibroblasts exhibited large decreases in their average motility after 100 mJ/cm^2^ UV-B (*P* < 0.001).

### MSC surface marker expression and morphology are unaffected by UV-B irradiation

**Figure 3:**
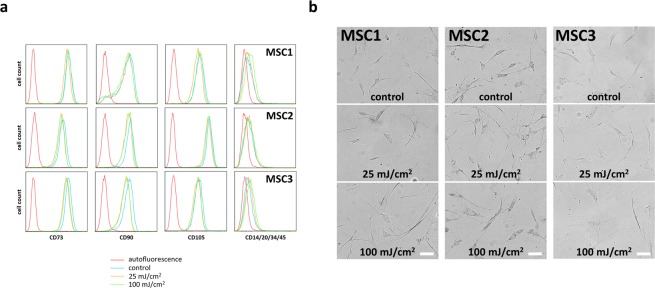
MSC morphology and surface marker expression are not affected by UV-B irradiation. (**a**) Flow cytometry histograms of both positive (CD73, CD90 and CD105) and negative (CD14, CD20, CD34 and CD45) MSC surface markers at 96 hours after UV-B irradiation. (**b**) Cellular morphology at 35 hours after UV-B exposure in MSCs and HS68 (scale bar 200 µm).

Surface marker expression of MSCs 96 hours after UV-B irradiation was examined by flow cytometry. Both 25 mJ/cm^2^ and 100 mJ/cm^2^ did not affect the expression of positive stem cell surface markers CD73, CD90 and CD105 in all tested MSC samples (Fig. 3a). Similarly, the lack of expression of the hematopoietic markers CD14, CD20, CD34 and CD45 remained unchanged after UV-B exposure.

Morphology of MSCs and dermal fibroblasts appeared largely unaltered 35 hours after UV irradiation and especially no morphological signs of apoptosis could be detected (Fig. 3b).

### Differentiation capacity of MSCs is maintained after UV-B irradiation

**Figure 4:**
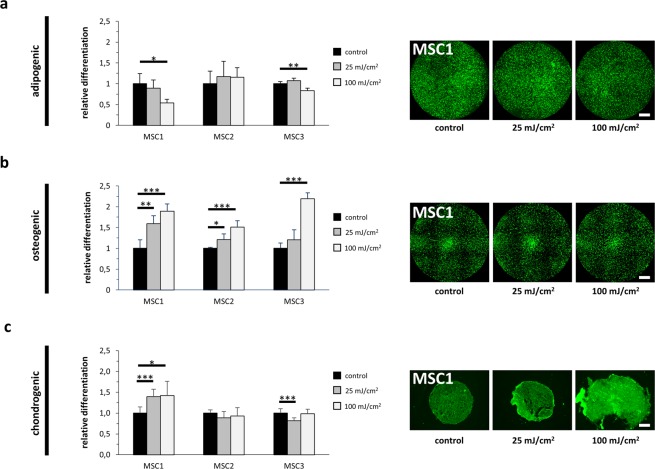
UV-B irradiation does not impair the differentiation capacity of MSCs. (**a**) BODIPY (493/503) staining of lipid droplet showing adipogenic differentiation of MSCs after exposure to UV-B light (scale bar 1000 µm). (**b**) OsteoImage™ staining for quantification of hydroxyapatite formation in differentiated MSCs after UV-B treatment (scale bar 1000 µm). (**c**) Aggrecan staining demonstrating chondrogenic differentiation in untreated and UV-B-exposed MSCs (scale bar 100 µm). *P < 0.05, **P < 0.01, ***P < 0.001. Bars show mean values, while error bars represent standard deviation, n ≥ 4.

Adipogenic, osteogenic and chondrogenic differentiation capabilities of MSCs were found to be largely maintained after UV-B irradiation as quantified by immunocytochemical stainings. 25 mJ/cm^2^ UV-B did not reduce the adipogenic differentiation in any MSC preparation, while 100 mJ/cm^2^ decreased adipogenic differentiation in MSC1 and MSC3, but not in MSC2 (*P* < 0.05 for MSC1, *P* < 0.01 for MSC3) (Fig. 4a, Supplementary Fig. 1A). Interestingly, both low-dose and high-dose UV-B irradiation increased the osteogenic differentiation potential of MSC1 and MSC2 (*P* < 0.01 for MSC1 after 25 mJ/cm^2^, *P* < 0.05 for MSC2 after 25 mJ/cm^2^, *P* < 0.001 for MSC1 and MSC2 after 100 mJ/cm^2^) (Fig. 4b, Supplementary Fig. 1B). More heterogeneous results were observed regarding chondrogenic differentiation after UV-B exposure. While chondrogenic differentiation increased in MSC1 (*P* < 0.001 at 25 mJ/cm^2^, *P* < 0.05 at 100 mJ/cm^2^), MSC3 revealed slightly reduced chondrogenic differentiation ability only after low-dose UV-B irradiation (*P* < 0.001) (Fig. 4c, Supplementary Fig. 1C), while MSC2 were unaffected at both doses.

### Low-dose UV-B irradiation leads to G2/M arrest in MSCs

Flow cytometry analyses were carried out to analyze cell cycle distribution after UV-B exposure. Low-dose irradiation caused an accumulation of cells in the G2/M phase 24 hours after treatment, which persisted at later time points (Supplementary Fig. 2). The cell cycle distribution of MSCs after high-dose irradiation appeared more heterogeneous and after 96 hours, only MSC1 and MSC3 exhibited an increase in their G2 phase population (*P* < 0.001), whereas MSC2 revealed a small decrease (*P* < 0.05). HS68 fibroblasts clearly showed a dose-dependent G2/M phase accumulation upon UV irradiation with 100 mJ/cm^2^. This pronounced and lasting G2/M arrest involves more than 50% cells of the population compared to 23.3% in untreated controls (*P* < 0.001), while low-dose UV-B only causes a minor increase.

### UV-B irradiation induces low apoptosis rates in MSCs

**Figure 5:**
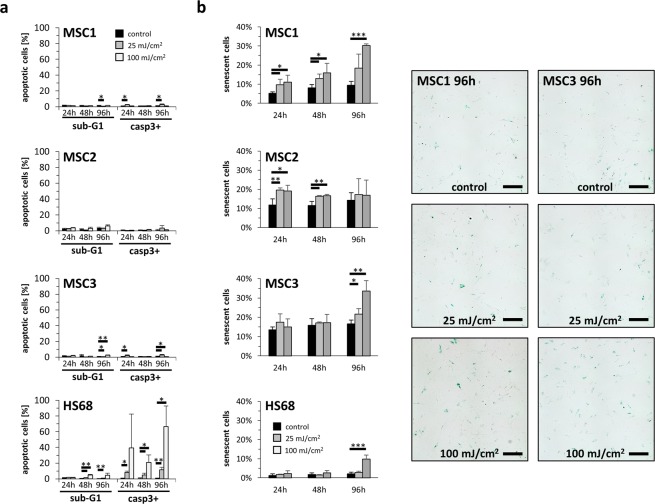
MSCs exhibited low apoptosis levels after UV-B irradiation. (**a**) Percentage of apoptotic cells assessed by sub-G1 population and caspase-3 activation in MSCs and HS68 at 24, 48 and 96 hours after irradiation with UV-B light. Data are shown as mean ± standard deviation, n = 3. (**b**) Relative β-galactosidase staining intensity after 25 mJ/cm^2^ or 100 mJ/cm^2^ UV-B irradiation. Representative images show β-galactosidase expression as cellular senescence marker 96 hours after UV-B treatment (scale bar 200 µm). *P < 0.05, **P < 0.01, ***P < 0.001. Data are mean ± standard deviation, n = 4.

Sub-G1 population and caspase-3 activation were measured to quantify apoptosis induction after UV-B treatment. Overall apoptosis rates remained low in all analyzed MSC samples with levels below 10% (Fig. 5a). In contrast, dermal fibroblasts exhibited increased apoptosis rates after both low-dose (25 mJ/cm^2^) and high-dose (100 mJ/cm^2^) UV-B irradiation, and more than 50% of HS68 cells were apoptotic 96 hours after 100 mJ/cm^2^ as determined by caspase-3 activation (*P* < 0.05).

To investigate cellular senescence after UV-B exposure, β-galactosidase stainings were conducted. 100 mJ/cm^2^ UV-B increased senescence rates in MSC1 and MSC2 after 24 hours (*P* < 0.05) (Fig. 5b). 96 hours after 100 mJ/cm^2^ UV treatment, both MSC1 and MSC3 showed elevated senescence rates with more than 30% senescent cells (*P* < 0.001 for MSC1, *P* < 0.01 for MSC3). In contrast, dermal fibroblasts exhibited low senescence levels without increases 24 and 48 hours after UV treatment. Only after 96 hours, high-dose UV-B irradiation led to augmented senescence rates in HS68 (*P* < 0.001).

### MSCs exhibit a more efficient CPD repair than dermal fibroblasts

**Figure 6:**
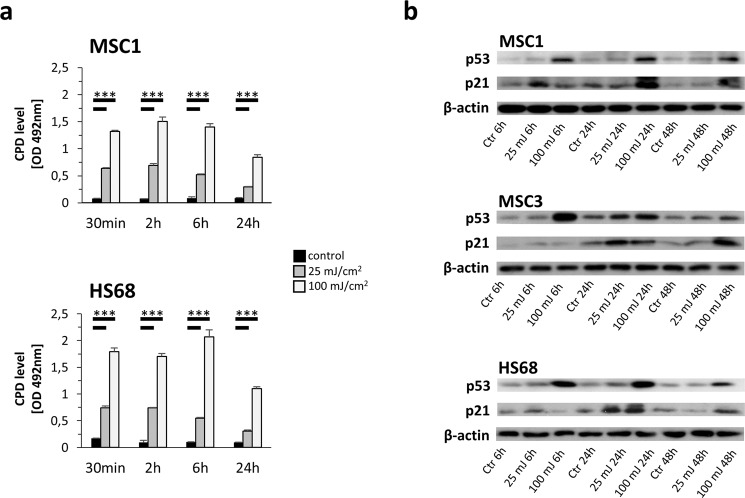
MSCs efficiently repair UV-B-induced CPDs. (**a**) Quantification of CPDs in MSC1 and HS68 at different time points after UV-B irradiation with 25 mJ/cm^2^ or 100 mJ/cm^2^ measured by ELISA. Mean ± standard deviation is shown, n = 3. (**b**) Western Blot analyses of p53 and p21 at different time points after UV-B exposure. Bands were cropped from individual gels, and actin controls were carried out for each gel to check for equal protein loading. *P < 0.05, **P < 0.01, ***P < 0.001.

Repair of UV-B-induced CPDs was investigated using ELISA analyses. CPD levels were found elevated already 30 minutes both after 25 mJ/cm^2^ and 100 mJ/cm^2^ UV irradiation in MSC1 and HS68 and remained stably elevated until the 6-hour timepoint (*P* < 0.001) (Fig. 6a). 24 hours after UV-B irradiation, CPD levels were significantly increased compared to untreated controls both for MSC1 and HS68 cells (*P* < 0.001). However, MSCs exhibited about 25% lower CPD levels than HS68 fibroblasts 24 hours after 100 mJ/cm^2^.

### UV-B irradiation increases expression of p53 and p21 in MSCs

Western blot analyses revealed p53 stabilization and increased expression of p21 in MSCs and HS68 fibroblasts upon UV-B irradiation especially after high UV-B doses (Fig. 6b). P53 levels were elevated already at 6 hours after irradiation and showed a dose-dependent response with higher levels after 100 mJ/cm^2^. Whereas prolonged p53 stability and increased p21 levels were observed even 24 and 48 hours after irradiation with 100 mJ/cm^2^ UV-B, 25 mJ/cm^2^ UV-B resulted in similar p53 and p21 protein levels especially at later timepoints.

## Discussion

While MSCs have shown beneficial effects regarding the regeneration of UV-induced skin damage, the effects of UV irradiation on MSCs themselves are largely unknown. Here, we elucidated the influence of UV-B irradiation on the survival and functional abilities of MSCs as well as mechanisms how MSCs deal with UV-B-induced DNA damage. We could show for the first time that human MSCs are relatively resistant to UV-B treatment and largely preserve their stem cells’ characteristics.

MSCs have been shown to reduce UV-induced skin damage by secreting paracrine factors including keratinocyte growth factor (KGF), basic fibroblast growth factor-1 (FGF-1) and vascular endothelial growth factor (VEGF), thereby promoting collagen and fibronectin production of dermal fibroblasts^23,24,30^. Preclinical and early clinical studies have demonstrated beneficial effects of MSC-based therapies for psoriasis and atopic dermatitis^25,26^. In a phase I/IIa study, patients with atopic dermatitis were treated with MSCs leading to a 50% reduction of the Eczema Area and Severity Index (EASI) score in the majority of patients^25^. Based on these encouraging results, a phase III trial now evaluates the efficacy of MSCs for atopic dermatitis (NCT03269773). The favorable results of MSC therapies for autoimmune skin disorders were mainly attributed to MSCs’ paracrine effects and their immunomodulatory impact, e.g. through prostaglandin E2 and transforming growth factor-β1 (TGF-β1)^31^.

As the stem cells’ ability to aid the regeneration of UV-damaged skin requires intact migratory functions, the observed reduction of cellular velocity after high-dose UV-B irradiation may reduce their regenerative effects. The decreased cellular motility in MSCs is in contrast to melanoma cells which exhibit increased cellular motility after UV-B irradiation via autocrine interleukin-8 (IL-8) secretion^32^. However, only low UV-B doses up to 30 mJ/cm^2^ were used in this study.

Data about the influence of UV-B irradiation on the adhesion ability of MSCs has been lacking so far. Although general adhesion ability of MSCs was maintained after UV-B irradiation, heterogenous results for MSCS derived from different donors were observed in our study. MSCs isolated from old donors have been shown to be more susceptible to ROS leading to reduced integrin expression, impaired adhesion ability and reduced engraftment rates in a myocardial infarct model; however, the donor’s age of our MSC preparations was quite homogeneous and ranged between 20 years and 32 years^33^. UV-B treatment has shown to inhibit intercellular adhesion molecule 1 (ICAM-1) induction by γ-interferon in keratinocytes at early time points, while it results in ICAM-1 induction at later time points beginning 48 hours after exposure^34^. A similar biphasic response was observed for melanocytes and melanoma cells: Cytokine-induced ICAM-1 synthesis was inhibited within the first 16 hours and increased between 48 and 96 hours after UV-B treatment^35^. The heterogeneous responsiveness of MSCs adhesion ability towards UV-B irradiation may be one possible explanation for different results upon therapeutic UV irradiation in autoimmune skin diseases.

Proliferation and cellular viability *in vitro* can partly predict the regenerative capacity of MSCs *in vivo*, and the increased proliferation and viability values compared to dermal fibroblasts are a promising indicator of an intact regenerative capacity^36^. Interestingly, metabolic viability determined by MTS assays was relatively well preserved after high UV-B doses up to 1500 mJ/cm^2^ UV-B irradiation, while clonogenic survival rates were below 5% compared to untreated controls at 200 mJ/cm^2^. Obviously, cellular reproductive death occurs at much lower UV doses than impairment of metabolic viability. Clonogenic survival assays are commonly used for evaluation of radiation sensitivity and determine the cells’ ability to undergo multiple cell divisions, whereas MTT and MTS assays are rather applied to study cellular chemosensitivity by measuring cell proliferation and intact mitochondrial respiration^37,38^. A similar discrepancy between clonogenic survival and metabolic viability of MSCs was observed for some chemotherapeutic agents such as paclitaxel and topoisomerase inhibitors^39,40^.

MSCs exert their regenerative effects via differentiation into functional cells and the creation of a protective microenvironment. Therefore, their preserved differentiation capacity is a further surrogate parameter for sufficient regenerative abilities of MSCs after UV irradiation. Our data suggest that endogenous dermal MSCs may preserve their regenerative abilities after single dose UV-B exposure. However, it is challenging to investigate whether multiple exposures to UV-B radiation induce different effects on MSCs, as they exhibit a short culturing time due to premature senescence *in vitro*^41^.

Previous studies have demonstrated that MSCs exhibit varying sensitivities to different DNA-damaging agents; however, a relative resistance has been shown for the majority of DNA-targeting chemotherapeutical agents^39,42–45^. Efficient DNA damage repair and high expression of anti-apoptotic proteins contribute to the cells’ ability to evade apoptosis^46,47^. The observed low apoptosis levels after UV-B irradiation are consistent with previous reports showing that MSCs may evade apoptosis induction by undergoing premature senescence^48^. Accordingly, we detected significantly increased β-galactosidase expression in two MSC samples. Senescent MSCs exhibit a reduced regenerative potential with a compromised immunoregulatory capacity^49^. Whether UV-B-mediated senescence induction in MSCs may impair the regenerative ability of MSCs *in vivo* needs to be investigated in further studies.

Previous studies have examined the effects of varying UV-B doses on the stability, modifications and activity of the tumor suppressor p53^50^. P53 protein levels are generally low due to constant degradation via ubiquitin-dependent proteolysis^51^. While low UV-B doses generally result in fast but transient p53 accumulation, higher UV-B doses lead to delayed but prolonged p53 protein level increase^52^. However, both doses used in our study (25 mJ/cm^2^ and 100 mJ/cm^2^) are considerably lower than required for sustained p53 accumulation (e.g. 350 mJ/cm^2^ UV-B were used in the study by Latonen *et al*. to induce a prolonged p53 increase^52^). In line with this, p53 levels were comparable between untreated and UV-irradiated MSC3 cells and only slightly elevated in UV-irradiated MSC1 48 hours after UV exposure. P21 is a downstream effector of p53 and inhibits several cyclin-dependent kinases (CDKs), finally leading to cell cycle arrest^53^. Additionally, p21 has a crucial role in senescence induction and has been shown to protect from p53-mediated apoptosis^54,55^. Accordingly, p21 levels were considerably increased in MSCs 24 and 48 hours after high dose (100 mJ/cm^2^) UV-B treatment which could at least partly explain increased senescence and low apoptosis rates of MSCs.

Nucleotide excision repair (NER) has been established as the main repair mechanism of UV-induced photoproducts such as CPDs and 6-4PPs^56^. Several readouts including T4 endonuclease V activity, UV-induced DNA repair synthesis or CPD repair measurements have been used to quantify NER after UV-B irradiation^57,58^. In our study, determination of NER activity was based on quantifying CPD levels after treatment. In accordance with previous reports demonstrating an effective NER activity of MSCs, we found that MSCs were able to repair UV-B-induced CPDs more efficiently than dermal fibroblasts^59^. In line with these findings, another study reported efficient DNA glycosylase activity in cultured adipose tissue-derived MSCs^60^.

In our dataset, human MSCs derived from bone marrow samples were used. It is conceivable that our results are also valid regarding skin-derived MSCs as both types have been demonstrated to show similar cellular morphology, surface markers expression and differentiation ability^61^. However, this hypothesis needs corroboration in further studies.

While several results were highly consistent after UV-B treatment for all tested MSC samples, including increased survival rates, impaired cellular velocity, stable surface marker expression, elevated osteogenic differentiation potential and low apoptosis levels, some results in our dataset revealed a considerable heterogeneity between individual MSCs. Regarding the increased senescence rates for MSC2 after UV-B exposure, we cannot rule out a potential impact of the donor’s age on the induction of cellular senescence. Some studies have shown a link between donor’s age and senescence levels *in vitro*, so that the increased age of donor #2 (32 years) compared to the other donors (20 years for donor#1, 25 years for donor#3) may contribute to the different senescence rate after UV-B treatment^62^. The known heterogeneity of MSCs derived from different donors may also need to be taken into account for the application of MSC-based treatments for skin diseases.

Prior to routine clinical application of MSC-based therapies for UV-induced skin damage or autoimmune skin diseases, any pro-tumorigenic potential especially for skin cancer must be thoroughly ruled out. It has been previously shown that growth of B16 melanoma cells was enhanced in the presence of co-injected MSCs, especially when MSCs were pre-incubated with interferon-γ and tumor necrosis factor-α which led to increased expression of the immunosuppressive enzyme inducible nitric oxide synthase (iNOS) in MSCs^63^. As iNOS inhibition abrogated the tumor-promoting effects of MSCs, the immunosuppressive abilities of MSCs may be one reason for the observed pro-tumorigenic effects of these cells in the model used. However, other studies have reported contrary effects of MSCs on melanoma cells such as reduction of proliferation *in vitro* and inhibition of tumor growth *in vivo*^64^.

Besides the known DNA-damaging potential, UV-B irradiation is also able to generate ROS, leading to oxidative damage and skin carcinogenesis as a potential long-term result^65^. Although we have not examined the anti-oxidative capacity of MSCs after UV-B treatment, several publications have reported the stem cells’ ability to efficiently inactivate ROS due to high glutathione and superoxide dismutase levels^66^. The efficient antioxidative capacity of MSCs may contribute to the observed UV-B resistance of MSCs; however, further experiments are needed to elucidate the role of the antioxidative capacity in terms of the stem cells’ UV-B response.

Ambient UV exposure comprises mainly UV-A irradiation at a wavelength of 315 to 400 nm which is, compared to UV-B irradiation, less intense but penetrates more deeply^67^. A limited number of studies examined the effects of UV-A irradiation on MSCs and revealed reduced adipogenic differentiation capacity but unchanged gene expression after UV-A-exposure^68,69^. UV-A acts mainly via indirect and ROS-mediated DNA damage, and the DNA-damaging effect of UV-A is less pronounced than that of UV-B. Therefore, our results may not be completely transferrable to the response of MSCs to UV-A irradiation.

In summary, our findings indicate a UV-B resistant phenotype of MSCs which may contribute to MSCs’ ability to attenuate UV-B-induced skin injuries. Considering the UV-protective and immunomodulatory properties of MSCs, our data may warrant further analyses regarding combination studies of MSCs and UV-B irradiation for the treatment of autoimmune skin diseases.

## Materials and Methods

### Cell culture

Human MSCs were isolated from the bone marrow of three healthy donors as described before (MSC1: male donor (20 years old), MSC2: male donor (32 years old), MSC3: male donor (25 years old)^70^. Informed consent was obtained prior to bone marrow aspiration, and this investigation was approved by the Heidelberg University ethics committee (#S-384/2004). Human HS68 dermal fibroblasts were purchased from the ATCC (Manassas, USA). Cells were maintained at 37 °C in a humidified incubator with 5% CO_2_. Mesenchymal Stem Cell Growth Medium (Lonza, Basel, Switzerland) was used for culturing MSCs, while HS68 cells were grown in Dulbecco’s Modified Eagle’s Medium (Biochrom, Berlin, Germany) supplemented with 10% fetal bovine serum. All analyses of this study were performed in accordance with the relevant guidelines and regulations.

### UV-B irradiation

UV-B irradiation was performed using a Waldmann UV181BL source (Waldmann, Villingen-Schwenningen, Germany) with an output range of 280–320 nm wavelength. For each treatment, exact UV doses were measured with an UV detector (Waldmann). As the initial UV-B broadband dose used in psoriasis treatment is between 20 and 60 mJ/cm^2^, and minimal erythema dose normally ranges between 80 and 240 mJ/cm^2^, a low-dose (25 mJ/cm^2^) and a high-dose (100 mJ/cm^2^) treatment group were used in the experiments^71,72^, except of assays for clonogenic survival, proliferation and viability where doses up to 1500 mJ/cm^2^ where used.

### Clonogenic, proliferation and viability assays

For clonogenic survival assays, between 400 and 1800 cells were plated in 6-well plates prior to treatment and allowed to grow for 14 days. Colonies were fixed with 25% acetic acid in methanol and stained with crystal violet solution. Colonies containing more than 50 cells were counted using an inverted Leica DM IL microscope (Leica Microsystems, Wetzlar, Germany), and the survival fraction was calculated as follows: (#colonies/#plated cells)_treated_/(#colonies/#plated cells)_untreated_. Experiments were performed with three biological triplicates.

To investigate the proliferation activity and viability after UV-B irradiation, between 3 × 10^4^ and 4 × 10^4^ cells were seeded in 6-well plates, and UV-B irradiation was performed 24 hours later. At 96 hours after irradiation, cells were harvested and stained with trypan blue to count viable cells using a Neubauer chamber.

At 96 hours after UV-B irradiation, cellular viability was assessed by the MTS assay (Promega, Madison, USA) using the tetrazolium compound 3-(4,5-Dimethylthiazol-2-yl)-5-(3-carboxymethoxyphenyl)-2-(4-sulfophenyl)-2H-tetrazolium. 24 hours prior to irradiation, 2 × 10^3^ cells/well were plated in a 96-well plate. Following incubation with 20 µL MTS solution for 2 hours, light absorbance was measured at 492 nm using a microplate reader (Tecan, Crailsheim, Germany).

### Cell adhesion measurements

Cells were grown in Petri dishes to a confluence of 70% prior to UV-B irradiation. Immediately after treatment, 100 cells/well were seeded in 96-well plates, and the number of attached cells was counted at different time points. The ratio between attached and seeded cells was calculated for each time point to determine the adhesion rate.

### Cellular velocity assays

Cellular velocity was measured by time-lapse microscopy. 2 × 10^4^ were plated in Petri dishes (10 cm diameter) prior to UV-irradiation. Time-lapse microscopy was conducted on the Keyence BioRevo9000 microscope (Keyence, Neu-Isenburg, Germany) fitted with an incubator box at 37 °C and 5% CO_2_. Manual single-cell tracking with ImageJ software (National Institutes of Health, Bethesda, USA) was used for quantification, and at least 10 cells/well from three randomly chosen fields-of-view were tracked.

### Surface marker expression

Cells were plated in Petri dishes (diameter 10 cm) to a confluence of 70% and UV-irradiated. At 96 hours after UV-B irradiation, cells were harvested and fixed with 3% paraformaldehyde in PBS. MSC surface marker expression was analyzed using the MSC Phenotyping Kit (Miltenyi Biotec, Bergisch-Gladbach, Germany) following the manufacturer’s instructions. Surface marker expression was determined on a FACSCanto^TM^ flow cytometer (BD, Heidelberg, Germany), and data analysis was performed with FlowJo 7.6.5 software (FlowJo LLC, Ashland, USA).

### MSC differentiation analyses

For adipogenic differentiation, 3 × 10^4^ cells were plated on glass cover slips in 24-well plates and irradiated with UV-B. To induce adipogenic differentiation, cell culture medium was replaced by STEMPRO® Adipogenesis differentiation medium (Gibco, Grand Island, NY, USA) and renewed twice per week. After 14–21 days, specimens were stained using 1 μg/mL BODIPY (493/503) (Life Technologies, Darmstadt, Germany), and nuclei were counterstained with 2 mM Hoechst33342 (Sigma, Steinheim, Germany).

For osteogenic differentiation, 25000 cells were plated on glass cover slips in 24-well plates and UV-irradiated 24 hours later. STEMPRO® Osteogenesis differentiation medium (Gibco) was used to induce osteogenic differentiation. For quantification, specimens were incubated with OsteoImage™ Staining Reagent (Lonza) according to the manufacturer’s instructions.

Chondrogenic differentiation was performed using the STEMPRO® Chondrogenesis Differentiation Kit (Gibco). After UV-B irradiation, 1 × 10^5^ cells/well were plated in 96-well plates in order to induce spheroids. 21 days later, spheroids were fixed with 4% paraformaldehyde in PBS, frozen at −20 °C and sectioned on a cryomicrotome. Sections were incubated with 0.3% Triton X-100, 1% BSA and 10% normal donkey serum in PBS prior to incubation with an antibody against human aggrecan (1:10, R&D Systems, Minneapolis, MN, USA) and counterstaining with an Alexa488-coupled secondary antibody (1:200, Donkey Anti-Goat; Abcam, Cambridge, UK).

For all differentiation experiments, fluorescence images were obtained from with a Keyence BioRevo9000 microscope, and staining intensities were normalized to cell numbers.

### Cell cycle and apoptosis measurements

At various time points after UV-B irradiation, cells were harvested and fixed with 3% paraformaldehyde in PBS before permeabilization using ice-cold 70% ethanol. Cells were then washed thrice with 0.5% bovine serum albumin (BSA) in PBS. To assess apoptosis, cells were incubated with an antibody against activated caspase-3 (1:20, BD Pharmingen, San Diego, CA, USA) for 1 hour. After centrifugation at 200x g, cells were resuspended in 1 µg/mL DAPI/PBS staining reagent. Cell cycle distribution and apoptosis rate were assessed with a LSR II flow cytometer (BD), and data analysis was carried out using FlowJo 7.6.5 as reported before^73^. Experiments were performed with three replicate samples.

### Senescence analyses

2 × 10^3^ cells were seeded on each glass cover slip in a 24-well plate prior to UV-B treatment. At various time points after irradiation, cells were fixed, and β-galactosidase activity was measured using the Senescence β-galactosidase Staining Kit (Cell Signaling Technology, Leiden, Netherlands) following the manufacturer’s instructions. Images were obtained with a Keyence BioRevo9000 microscope, and assessment of β-galactosidase-positive cells was performed using ImageJ.

### CPD ELISA assays

Cells were UV-B irradiated, and at different time points after treatment, DNA was isolated using the QIAamp® DNA Mini Kit (Qiagen, Hilden, Germany). Cells were incubated with proteinase K (activity: 600 mAu/mL) and lysis buffer at 56 °C for 10 minutes, before 100% ethanol was added to the sample. DNA was purified using QIAamp® Mini spin columns, and DNA concentration was quantified by NanoDrop (Thermo Scientific, Wilmington, DE, USA). Analysis of CPD repair was performed using the High Sensitivity CPD ELISA Kit (Cosmo Bio Co., Tokyo, Japan) according to the manufacturers’ protocol. Briefly, DNA was coated to the plate, and after washing and blocking of non-specific antibody binding, wells were incubated with anti-CPD antibody (1:100, clone TDM-2). Following incubation with the biotinylated secondary antibody (1:100), streptavidin-peroxidase was added. Wells were incubated with O-phenylenediamine, and absorbance at 492 nm was determined using a SPECTROstar Nano microplate reader (BMG LABTECH, Ortenberg, Germany).

### Western blots

MSCs and HS68 dermal fibroblasts were grown in Petri dishes to a confluence of 80% and then exposed to UV-B radiation. Cells were harvested at various time points after treatment, and cell pellets were incubated in RIPA buffer for 20 minutes on ice. Protein samples were run on 12% tris-acetate gels and transferred to polyvinylidenedifluoride membranes (Millipore, Darmstadt, Germany). After blotting, membranes were incubated with antibodies against p53 (1:1000, Cell Signaling) and p21 (1:500, BD Pharmingen), while β-actin (1:500, MP Biomedical, Solon, OH, USA) was used as a loading control. After incubation and several washing steps, membranes were incubated with the HRP-conjugated secondary antibodies anti-mouse-HRP (1:1000, W402B, Promega) and anti-rabbit-HRP (1:1000, W401B, Promega). Western blots were visualized on X-ray films using a Luminol-based enhanced chemiluminescence (ECL) HRP substrate (Thermo Scientific™ SuperSignal™ West Dura Chemiluminescent Substrate) following the manufacturer’s instructions.

### Statistics

At least three experimental replicates were carried out to calculate mean values and standard deviations. Comparisons between control and treatment group were performed using unpaired, two-sided Student’s t-tests. P-values < 0.05 were considered significant.

## Supplementary information

Supplementary figures

## Data Availability

The datasets generated during and/or analyzed during the current study are available from the corresponding author on reasonable request.

## References

[1] Dawe RS (2003). A randomized controlled trial of narrowband ultraviolet B vs bath-psoralen plus ultraviolet A photochemotherapy for psoriasis. Br. J. Dermatol..

[2] Hofer A, Cerroni L, Kerl H, Wolf P (1999). Narrowband (311-nm) UV-B therapy for small plaque parapsoriasis and early-stage mycosis fungoides. Archives of Dermatology.

[3] Westerhof W, Nieuweboer-Krobotova L (1997). Treatment of vitiligo with UV-B radiation vs topical psoralen plus UV-A. Archives of dermatology.

[4] George S, Bilsland D, Johnson B, Ferguson J (1993). Narrow‐band (TL‐01) UVB air‐conditioned phototherapy for chronic severe adult atopic dermatitis. British Journal of Dermatology.

[5] AUBIN F (2003). Mechanisms involved in ultraviolet light‐induced immunosuppression. European journal of dermatology.

[6] Ozawa M (1999). 312-nanometer ultraviolet B light (narrow-band UVB) induces apoptosis of T cells within psoriatic lesions. Journal of experimental medicine.

[7] Lee HST, Kooshesh F, Saunder DN, Kondo S (1997). Modulation of TGF‐β1 production from human keratinocytes by UVB. Experimental dermatology.

[8] de Gruijl FR (2000). Photocarcinogenesis: UVA vs UVB. Methods Enzymol.

[9] Burnworth B (2007). The multi-step process of human skin carcinogenesis: A role for p53, cyclin D1, hTERT, p16, and TSP-1. European Journal of Cell Biology.

[10] You Y-H (2001). Cyclobutane pyrimidine dimers are responsible for the vast majority of mutations induced by UVB irradiation in mammalian cells. Journal of Biological Chemistry.

[11] Debacq-Chainiaux F, Leduc C, Verbeke A, Toussaint O (2012). UV, stress and aging. Dermatoendocrinol.

[12] Zuk PA (2002). Human adipose tissue is a source of multipotent stem cells. Mol Biol Cell.

[13] Bieback K, Kern S, Kluter H, Eichler H (2004). Critical parameters for the isolation of mesenchymal stem cells from umbilical cord blood. Stem Cells.

[14] Anker PS (2004). Isolation of mesenchymal stem cells of fetal or maternal origin from human placenta. Stem Cells.

[15] Toma JG (2001). Isolation of multipotent adult stem cells from the dermis of mammalian skin. Nature cell biology.

[16] Friedenstein A, Chailakhjan R, Lalykina K (1970). The development of fibroblast colonies in monolayer cultures of guinea‐pig bone marrow and spleen cells. Cell Proliferation.

[17] Dominici M (2006). Minimal criteria for defining multipotent mesenchymal stromal cells. The International Society for Cellular Therapy position statement. Cytotherapy.

[18] Liu CH, Hwang SM (2005). Cytokine interactions in mesenchymal stem cells from cord blood. Cytokine.

[19] Fang X, Neyrinck AP, Matthay MA, Lee JW (2010). Allogeneic human mesenchymal stem cells restore epithelial protein permeability in cultured human alveolar type II cells by secretion of angiopoietin-1. J. Biol. Chem..

[20] Liu H, Zhang J, Liu CY, Hayashi Y, Kao WW (2012). Bone marrow mesenchymal stem cells can differentiate and assume corneal keratocyte phenotype. J. Cell Mol. Med..

[21] Lopatina T (2014). Platelet-derived growth factor regulates the secretion of extracellular vesicles by adipose mesenchymal stem cells and enhances their angiogenic potential. Cell Communication and Signaling.

[22] Cahill EF, Kennelly H, Carty F, Mahon BP, English K (2016). Hepatocyte Growth Factor Is Required for Mesenchymal Stromal Cell Protection Against Bleomycin‐Induced Pulmonary Fibrosis. Stem cells translational medicine.

[23] Kim WS (2008). Evidence supporting antioxidant action of adipose-derived stem cells: protection of human dermal fibroblasts from oxidative stress. J Dermatol Sci.

[24] Kim WS, Park BS, Park SH, Kim HK, Sung JH (2009). Antiwrinkle effect of adipose-derived stem cell: activation of dermal fibroblast by secretory factors. J. Dermatol. Sci..

[25] Kim HS (2017). Clinical Trial of Human Umbilical Cord Blood‐Derived Stem Cells for the Treatment of Moderate‐to‐Severe Atopic Dermatitis: Phase I/IIa Studies. Stem cells.

[26] Chen H (2016). Treatment of psoriasis with mesenchymal stem cells. The American journal of medicine.

[27] Rühle A (2018). The Radiation Resistance of Human Multipotent Mesenchymal Stromal Cells Is Independent of Their Tissue of Origin. International Journal of Radiation Oncology*Biology*Physics.

[28] Nicolay NH (2013). Mesenchymal stem cells retain their defining stem cell characteristics after exposure to ionizing radiation. Int J Radiat Oncol Biol Phys.

[29] Rühle A (2017). Cisplatin radiosensitizes radioresistant human mesenchymal stem cells. Oncotarget.

[30] Son W-C, Yun J-W, Kim B-H (2015). Adipose-derived mesenchymal stem cells reduce MMP-1 expression in UV-irradiated human dermal fibroblasts: therapeutic potential in skin wrinkling. Bioscience, Biotechnology, and Biochemistry.

[31] Kim HS (2015). Human umbilical cord blood mesenchymal stem cell-derived PGE2 and TGF-beta1 alleviate atopic dermatitis by reducing mast cell degranulation. Stem Cells.

[32] Gebhardt C (2007). Ultraviolet-B irradiation enhances melanoma cell motility via induction of autocrine interleukin 8 secretion. Experimental Dermatology.

[33] Li L (2014). Aging Increases the Susceptivity of MSCs to Reactive Oxygen Species and Impairs Their Therapeutic Potency for Myocardial Infarction. PLOS ONE.

[34] Norris DA, Lyons MB, Middleton MH, Yohn JJ, Kashihara-Sawami M (1990). Ultraviolet radiation can either suppress or induce expression of intercellular adhesion molecule 1 (ICAM-1) on the surface of cultured human keratinocytes. J. Invest Dermatol..

[35] Kirnbauer R (1992). Modulation of intercellular adhesion molecule-1 expression on human melanocytes and melanoma cells: evidence for a regulatory role of IL-6, IL-7, TNF beta, and UVB light. J Invest Dermatol.

[36] Deskins DL (2013). Human mesenchymal stromal cells: identifying assays to predict potency for therapeutic selection. Stem Cells Transl Med.

[37] Franken NA, Rodermond HM, Stap J, Haveman J, van Bree C (2006). Clonogenic assay of cells *in vitro*. Nat. Protoc..

[38] van Meerloo, J., Kaspers, G. J. L. & Cloos, J. In *Cancer Cell Culture:* Methods *and Protocols* (ed Ian A. Cree) 237–245 (Humana Press, 2011).

[39] Munz F (2018). Human mesenchymal stem cells lose their functional properties after paclitaxel treatment. Sci Rep.

[40] Bosco DB, Kenworthy R, Zorio DA, Sang QX (2015). Human mesenchymal stem cells are resistant to Paclitaxel by adopting a non-proliferative fibroblastic state. PLoS One.

[41] Kern S, Eichler H, Stoeve J, Klüter H, Bieback K (2006). Comparative analysis of mesenchymal stem cells from bone marrow, umbilical cord blood, or adipose tissue. Stem cells.

[42] Nicolay NH (2016). Mesenchymal stem cells are sensitive to bleomycin treatment. Sci Rep.

[43] Nicolay NH (2016). Mesenchymal stem cells maintain their defining stem cell characteristics after treatment with cisplatin. Sci Rep.

[44] Rühle, A., Huber, P. E., Saffrich, R., Lopez Perez, R. & Nicolay, N. H. The current understanding of mesenchymal stem cells as potential attenuators of chemotherapy‐induced toxicity. *International journal of cancer* (2018). 10.1002/ijc.31619 29931767

[45] Ruhle A (2019). The Therapeutic Potential of Mesenchymal Stromal Cells in the Treatment of Chemotherapy-Induced Tissue Damage. Stem Cell Rev.

[46] Oliver L (2011). Distinct Roles of Bcl-2 and Bcl-Xl in the Apoptosis of Human Bone Marrow Mesenchymal Stem Cells during Differentiation. PLOS ONE.

[47] Chen MF (2006). The sensitivity of human mesenchymal stem cells to ionizing radiation. Int J Radiat Oncol Biol Phys.

[48] Ko E, Lee KY, Hwang DS (2012). Human umbilical cord blood-derived mesenchymal stem cells undergo cellular senescence in response to oxidative stress. Stem Cells Dev.

[49] Carlos Sepúlveda J (2014). Cell Senescence Abrogates the Therapeutic Potential of Human Mesenchymal Stem Cells in the Lethal Endotoxemia Model. STEM CELLS.

[50] Latonen L, Laiho M (2005). Cellular UV damage responses—Functions of tumor suppressor p53. Biochimica et Biophysica Acta (BBA) - Reviews on Cancer.

[51] Maki CG, Huibregtse JM, Howley PM (1996). *In vivo* ubiquitination and proteasome-mediated degradation of p53(1). Cancer Res.

[52] Latonen L, Taya Y, Laiho M (2001). UV-radiation induces dose-dependent regulation of p53 response and modulates p53-HDM2 interaction in human fibroblasts. Oncogene.

[53] Li Y, Jenkins CW, Nichols MA, Xiong Y (1994). Cell cycle expression and p53 regulation of the cyclin-dependent kinase inhibitor p21. Oncogene.

[54] Gorospe M (1997). p21(Waf1/Cip1) protects against p53-mediated apoptosis of human melanoma cells. Oncogene.

[55] Mirzayans R, Andrais B, Scott A, Murray D (2012). New Insights into p53 Signaling and Cancer Cell Response to DNA Damage: Implications for Cancer Therapy. Journal of Biomedicine and Biotechnology.

[56] Rechkunova, N. & Lavrik, O. In *Genome Stability and Human Diseases* 251–277 (Springer, 2010).

[57] Steurer B (2019). Fluorescently-labelled CPD and 6-4PP photolyases: new tools for live-cell DNA damage quantification and laser-assisted repair. Nucleic Acids Research.

[58] Kobayashi N (2001). Quantitation and visualization of ultraviolet-induced DNA damage using specific antibodies: application to pigment cell biology. Pigment Cell Res..

[59] Alves H (2010). A link between the accumulation of DNA damage and loss of multi-potency of human mesenchymal stromal cells. Journal of Cellular and Molecular Medicine.

[60] Hildrestrand GA, Duggal S, Bjoras M, Luna L, Brinchmann JE (2009). Modulation of DNA glycosylase activities in mesenchymal stem cells. Exp. Cell Res..

[61] Liu R, Chang W, Wei H, Zhang K (2016). Comparison of the Biological Characteristics of Mesenchymal Stem Cells Derived from Bone Marrow and Skin. Stem Cells Int.

[62] Gnani D (2019). An early-senescence state in aged mesenchymal stromal cells contributes to hematopoietic stem and progenitor cell clonogenic impairment through the activation of a pro-inflammatory program. Aging Cell.

[63] Han Z (2011). Immunosuppressive effect of bone marrow-derived mesenchymal stem cells in inflammatory microenvironment favours the growth of B16 melanoma cells. J. Cell Mol. Med..

[64] Zhang J (2017). Inhibitory effect and mechanism of mesenchymal stem cells on melanoma cells. Clin. Transl. Oncol..

[65] Nishigori C, Hattori Y, Toyokuni S (2004). Role of reactive oxygen species in skin carcinogenesis. Antioxid Redox Signal.

[66] Valle-Prieto A, Conget PA (2010). Human mesenchymal stem cells efficiently manage oxidative stress. Stem cells and development.

[67] D’Orazio J, Jarrett S, Amaro-Ortiz A, Scott T (2013). UV radiation and the skin. International journal of molecular sciences.

[68] Lee J, Jung E, Hyun J-W, Park D (2012). Ultraviolet A regulates the stemness of human adipose tissue-derived mesenchymal stem cells through downregulation of the HIF-1α via activation of PGE2–cAMP signaling. Journal of Cellular Biochemistry.

[69] Lee J (2010). Ultraviolet A regulates adipogenic differentiation of human adipose tissue-derived mesenchymal stem cells via up-regulation of Kruppel-like factor 2. Journal of Biological Chemistry.

[70] Nicolay NH (2016). Mesenchymal stem cells exhibit resistance to topoisomerase inhibition. Cancer Lett..

[71] Menter A (2010). Guidelines of care for the management of psoriasis and psoriatic arthritis: Section 5. Guidelines of care for the treatment of psoriasis with phototherapy and photochemotherapy. J. Am. Acad. Dermatol..

[72] Li YW, Chu CY (2007). The minimal erythema dose of broadband ultraviolet B in Taiwanese. J. Formos. Med. Assoc..

[73] Lopez Perez R (2019). DNA damage response of clinical carbon ion versus photon radiation in human glioblastoma cells. Radiother. Oncol..

