# Supplementary material for: Human mesenchymal stem cells are resistant to UV-B irradiation
Source: Sci Rep. 2019 Dec 27;9:20000. doi: 10.1038/s41598-019-56591-9 (PMC6934474; doi:10.1038/s41598-019-56591-9)
Supplement: Supplementary file 1 — Supplementary figures [file 41598_2019_56591_MOESM1_ESM.pdf]

**Human mesenchymal stem cells are resistant to UV-B irradiation**

Ramon Lopez Perez, Ph.D.[1†]; Jannek Brauer[1,2†]; Alexander Rühle, M.D.[1,2†]; Thuy Trinh[1,2]; Sonevisay Sisombath[1]; Patrick Wuchter, M.D.[3]; Anca-Ligia Grosu, M.D.[4]; Jürgen Debus, M.D., Ph.D.[1,2]; Rainer Saffrich, Ph.D.[3]; Peter E. Huber, M.D., Ph.D.[1,2*]; Nils H. Nicolay, M.D., Ph.D.[1,4*]

**Supplementary Figure 1. UV-B irradiation does not affect MSCs' differentiation ability.** (**A**) Representative images showing BODIPY (493/503) staining for adipogenic differentiation of MSC2 and MSC3 after exposure to UV-B light (scale bar 1000 µm). (**B**) Representative images of OsteoImage™ staining for osteogenic differentiation of MSC2 and MSC3 after UV-B treatment (scale bar 1000 µm). (**C**) Representative images of aggrecan staining demonstrating chondrogenic differentiation in MSC2 and MSC3 after treatment with UV-B (scale bar 100 µm).

**Supplementary Figure 2. Low-dose UV-B irradiation results in a G2/M arrest of MSCs.** (**A**) Cell cycle distribution of MSCs and dermal fibroblasts at 24, 48 and 96 hours after UV-B irradiation. *$P<0.05$, **$P<0.01$, ***$P<0.001$. Mean ± standard deviation is shown, n=3.

*Supplementary Figure 1*

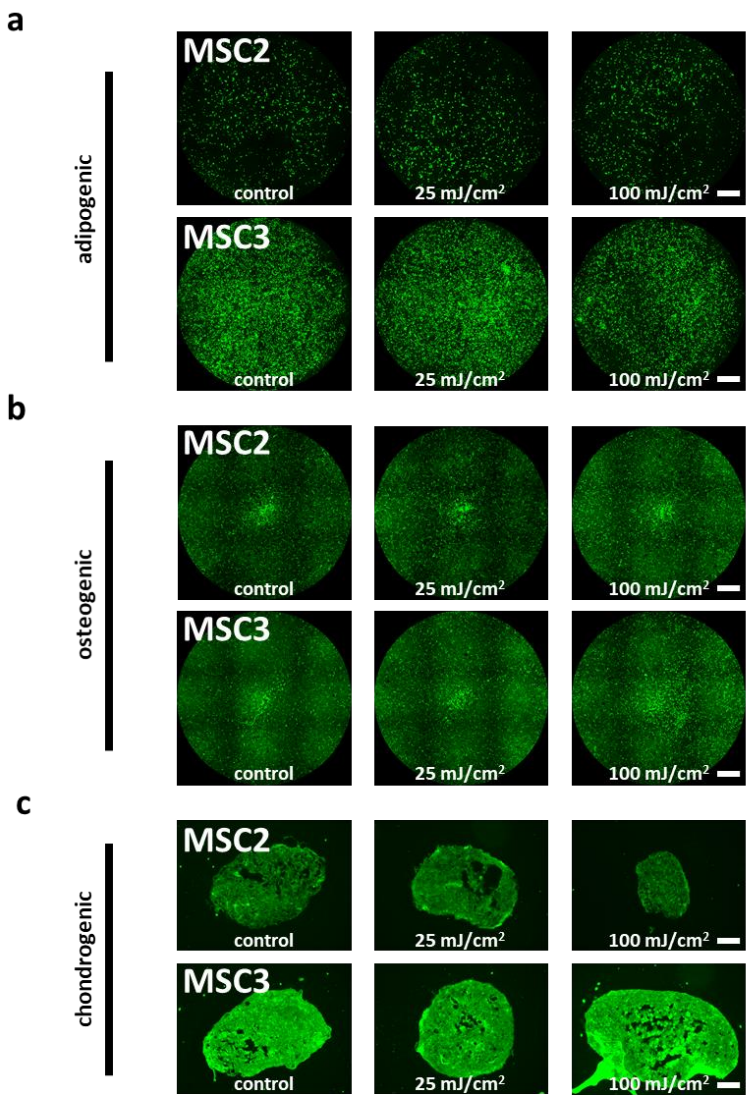

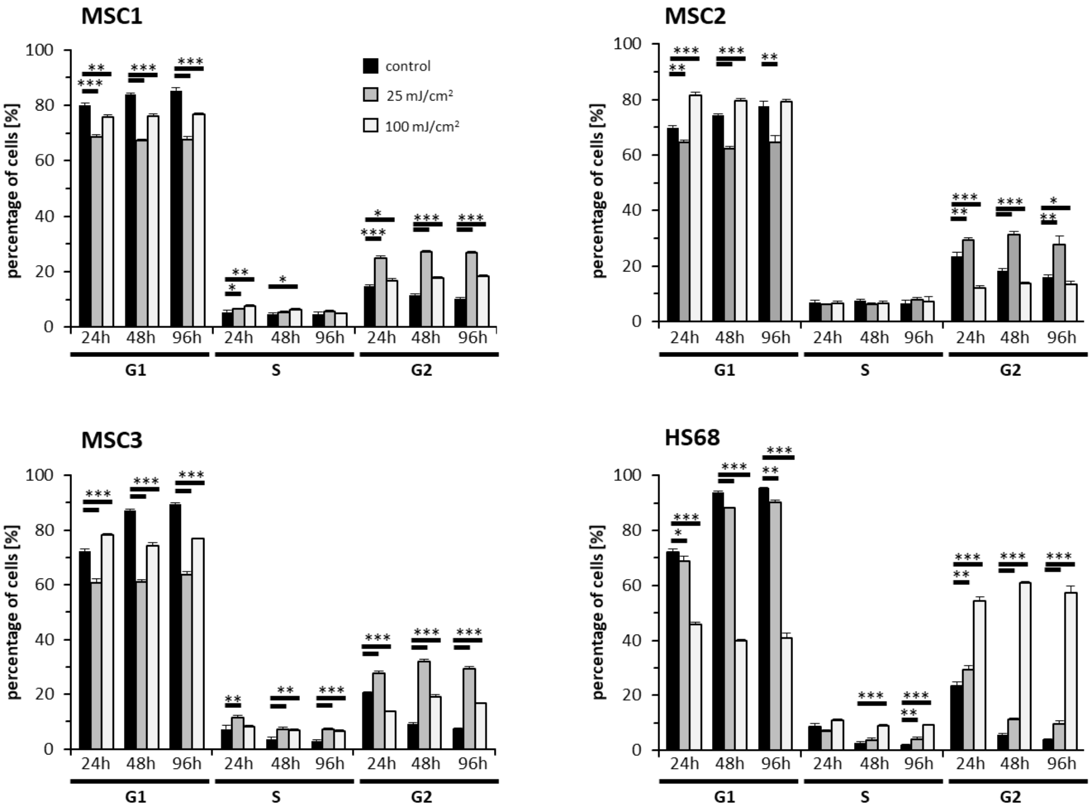

MSC1
MSC2
MSC3
HS68
control
25 mJ/cm²
100 mJ/cm²
percentage of cells [%]
0
20
40
60
80
100
24h
48h
96h
G1
S
G2
*
**
***
